# To what extent has doctoral (PhD) education supported academic nurse educators in their teaching roles: an integrative review

**DOI:** 10.1186/s12912-018-0273-3

**Published:** 2018-02-22

**Authors:** Carol Bullin

**Affiliations:** College of Nursing, University of Saskatchewan, Room 4338 E-Wing, Health Sciences Building, 104 Clinic Place, Saskatoon, SK S7N 2Z4 Canada

**Keywords:** Academic nurse educators, Doctoral preparation, Scholarship of teaching, Pedagogy, Undergraduate nursing education, Literature review

## Abstract

**Background:**

A doctoral degree, either a PhD or equivalent, is the academic credential required for an academic nurse educator position in a university setting; however, the lack of formal teaching courses in doctoral programs contradict the belief that these graduates are proficient in teaching. As a result, many PhD prepared individuals are not ready to meet the demands of teaching.

**Methods:**

An integrative literature review was undertaken. Four electronic databases were searched including the Cumulative Index to Nursing & Allied Health Literature (CINAHL), PubMed, Educational Resources Information Center (ERIC) and ProQuest. Date range and type of peer-reviewed literature was not specified.

**Results:**

Conditions and factors that influenced or impacted on academic nurse educators’ roles and continue to perpetuate insufficient pedagogical preparation include the requirement of a research focused PhD, lack of mentorship in doctoral programs and the influence of epistemic cultures (including institutional emphasis and reward system). Other factors that have impacted the academic nurse educator’s role are society’s demand for highly educated nurses that have increased the required credential, the assumption that all nurses are considered natural teachers, and a lack of consensus on the practice of the scholarship of teaching.

**Conclusions:**

Despite recommendations from nursing licensing bodies and a major US national nursing education study, little has been done to address the issue of formal pedagogical preparation in doctoral (PhD) nursing programs. There is an expectation of academic nurse educators to deliver quality nursing education yet, have very little or no formal pedagogical preparation for this role. While PhD programs remain research-intensive, the PhD degree remains a requirement for a role in which teaching is the major responsibility.

## Background

Professional health education has not adequately advanced in preparing health care workers to effectively meet the current and future expectations of the health care system [1]. Static curricula, lack of emphasis on pedagogy, and silo mentality are cited as barriers that have impeded changes necessary to professional education [1]. The Carnegie Foundation for the Advancement of Teaching’s national study on the transformation of nursing education [2], identified that nurses must have the abilities and skills to perform in multiple settings and contexts, within situations that are unclear, contextual, and dynamic. Addressing these changes are the responsibility of those providing nursing education, specifically academic nurse educators. Teachers of nursing education require both in-depth, discipline-specific and pedagogical knowledge to effectively meet the anticipated complexities of professional nursing practice [3].

A doctoral degree, either a doctorate of philosophy (PhD) or equivalent is currently the required academic credential for an academic nurse educator in a university setting. Interestingly, while approximately 80% of these graduates take a position in college/university teaching [4], the primary focus of PhD coursework is to develop research interests [5, 6]. Academics enter higher education with very high levels of knowledge in subjects or disciplines but no knowledge of teaching adults [7]. For this reason, few academic nurse educators are formally prepared for a teaching role [3, 8]. Skinner (as cited in Brightman) [2] best articulated the issue of the lack of formal teaching preparation in higher education:

It has been said that college teaching is the only profession for which there is no professional training, and it is commonly argues that this is because our graduate schools train scholars and scientists rather than teachers. We are more concerned with the discovery of knowledge than with its dissemination. (p.1).

Comprehending the issue around the lack of formal teaching preparation for academic nurse educators, necessitates a brief overview of both the current context of the discipline of nursing and nursing education, in addition to the historical development of academic preparation for the role of a nurse educator. This issue is common and not limited to either North America or to the discipline of nursing and nursing education [5, 9–11]. For academic nurse educators to provide quality learning experiences to nursing students, they require appropriate academic preparation that includes pedagogical knowledge, or simply stated, formal knowledge of ways to effectively communicate subject matter that fosters learning and ultimately, understanding. However, excellence in teaching is neither truly valued nor rewarded in many academic institutions [1, 12]. Academic scholarship is commonly defined strictly in terms of research. This undervaluing of teaching is also evident in nursing with the advent of the term *research* being incorporated into the scholarship of nursing. Prior to the mid-nineteenth century, teaching had been the primary focus of scholarship in higher education [13]. At this time, professional nursing faculties did not have the same emphasis on research as the broader university; however, as more nurses obtained PhDs, graduate programs were established, and programs of nursing research developed. The goal of nursing PhD programs was to prepare nurse scientists [14, 15], and as a result, teaching became a secondary activity.

Formal teaching preparation for nurses has declined significantly from 1976 in which 24% of nursing Masters’ programs graduates primary area of study was education (teaching), in contrast to 5.3% in 2004 [5]. This decline was due to an increased emphasis on preparing nurses to practice at an advanced clinical levels (i.e. nurse practitioners and clinical nurse specialists). The trend in advanced practice nursing has recently resurfaced in the United States with the entry level to advanced practice shifting from the masters to a doctoral degree [16]. This shift is evident in the proliferation of Doctor of Nursing Practice (DNP) programs in the United States. For example, in 2014, there were 5290 doctoral students enrolled in 130 PhD programs as compared to 18,352 doctoral students enrolled in 219 DNP programs [16]. However, while the DNP is an advanced clinical practice degree, many graduates are taking faculty positions for which they are not prepared [4, 17, 18]. It is well documented that programs leading to the Masters or doctoral degrees in Nursing does not prepare those nurses for many of the roles and responsibilities associated with academe [8, 19, 20].

In order to advance the scholarship of teaching, the AACN [5, 9, 21] identified the need to establish best practices in teaching, while the National League for Nursing (NLN) [21, 22] highlighted the development of nursing education theory and ultimately, educational mastery. However, faculty and administrators of graduate nursing programs have focused on developing nursing research and have continued to making little effort to prepare future faculty for teaching [3, 11, 23]. Therefore, if a PhD is required of academic nurse educators for a role in teaching and the focus of PhD preparation has traditionally privileged research and developing one’s role as a researcher, how will PhD preparation address the teaching component of the academic nurse educators’ role? Based on the information above, it is important to understand the state of the literature regarding a PhD requirement and the extent to which a PhD supports academic nurse educators in their teaching roles.

## Method

**Fig. 1:**
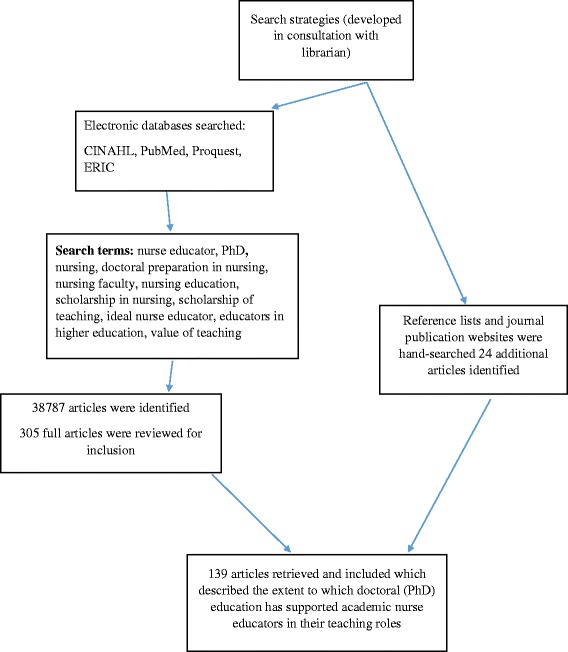
Search and Selection Process

A preliminary search, not restricted to English language literature, was done to determine what literature review strategy was most appropriate to answer the aim of the study. Based on the results of this search, an integrative literature review method [24] was selected. An integrative review allows for the integration of various types of literature and research methodologies. A keyword search of the literature was undertaken as of March 2017 using Cumulative Index to Nursing and Allied Health Literature (CINAHL), PubMed, Educational Resources Information Center (ERIC) and ProQuest databases. The search terms used included: “nurse educator”, “PhD”, “doctoral preparation in nursing”, “nursing faculty”, “nursing education”, “scholarship in nursing”, “scholarship of teaching”, “ideal nurse educator”, “educators in higher education”, and “value of teaching” in varying combinations. The population selected for this review were registered nurses that were either preparing or prepared, at the doctoral (i.e., PhD) level as a requirement for an academic nurse educator role in a university setting. The review process involved a search of the current literature, evaluation of the retrieved articles and synthesis of results. For inclusion in the integrative review all literature must have: 1) been available in English text, 2) focused on the experiences of academic nurse educators’ required doctoral preparation (in a university setting), 3) available in peer-reviewed outlets and, 4) no date restriction. Literature that referenced nurse educators in other than university settings was not included. The peer-reviewed literature was included or excluded based on whether or not it met the inclusion criteria. The search and selection process is outlined in Fig. 1.

## Results

In sum, a total of 139 peer-reviewed works were retrieved and included in this review, relative to the experience of nurse educators’ in preparing or prepared, at the doctoral level for a role in academe. Most were published in the United States (*n* = 126); other countries included Canada (*n* = 9), Australia (*n* = 3), Taiwan (n = 1), Slovania (n = 1) and, United Kingdom (n = 1). The date range is from 1990 to present. Types of peer-reviewed literature included research studies qualitative design (*n* = 21), quantitative design (*n* = 8), and mixed method design (*n* = 4); however, discussion papers and reports contributed much of the literature reviewed. A summary of these results are illustrated in Table 1 Summary of peer-reviewed literature and organized according to the themes and subthemes identified from the literature search.

**Table 1:** Summary of peer-reviewed literature

Author	Year	Theme/*subtheme*	Peer-reviewed literature	Literature content
Adams [62]	2011	Theme 1: Nurse Educator Expectations	Report	Expectations for preparing future faculty
AACN [5]	2005		Report	Nursing education faculty
Anderson [37]	2008		Qualitative, semi-structured interviews (*n* = 18)	Transitioning from a clinical expert to novice educator
Austin [51]	2002		Discussion paper	Future faculty preparation
Bass [98]	2006		Book	Re-examining doctoral education
Bartels [94]	2007		Discussion paper	Role preparation for the academy (scholarship)
Benner et al. [3]	2010		Book	Transforming nursing education
Bergner et al. [110]	2010		Discussion paper	Teaching training for PhD students
Booth et al. [8]	2016		Discussion paper	Formal pedagogical preparation required for nurse educators
Bok [135]	2013		Commentary	Preparing PhD students to teach
Bogo [136]	2010		Discussion paper	Preparing doctoral students to teach
Brightman [2]	2009		Discussion paper	Teaching doctoral students \how to teach
Cooley et al. [111]	2015		Qualitative, hermeneutical, phenomenology (*n* = 7)	Facilitators & barriers to nurse educator practice development
Diekelmann [63]	2005		Discussion paper	New pedagogies to transition nursing practice
Diekelmann [39]	2003		Book	New pedagogies for health professionals
Dreifeurst et al. [112]	2016		Sequential, explanatory, descriptive survey (*n* = 548)	PhD preparation for nurse faculty
Edwardson [6]	2004		Discussion paper	Shortcomings & relevance of the PhD
Fang et al. [56]	2016		Cross sectional questionnaire *(n* = 933)	Barriers & facilitators to nurse faculty roles
Fiedler et al. [11]	2015		Qualitative, in-depth interviews (*n* = 8)	Faculty preparation education course
Findlow [124]	2012		Discussion paper	Professional academic identity in nursing
Gaff [120]	2002		Discussion paper	Doctoral preparation for a faculty career (Preparing Future Faculty Program PFF)
Ivey [19]	2007		Discussion paper	Formal pedagogical preparation of nurse educators
Ironside [92]	2006		Editorial	Pedagogical preparation for nurse educators
Ironside [95]	2005		Qualitative hermeneutical (*n* = 45)	Pedagogical preparation for nurse educators
Jackson et al. [103]	2011		Sequential, exploratory mixed methods (*n* = 24)	PhD requirement for academic nursing
Johnsen-Crawley [25]	2004		Discussion paper	Teacher preparation models
Johnson-Farmer et al. [61]	2009		Qualitative, grounded theory (*n* = 17)	Teaching excellence as a dynamic process
Kalb et al. [137]	2012		Qualitative, online survey (*n* = 76)	Developing leadership in nursing
Kwiram [46]	2006		Book	Re-examination of doctoral education
Lancet Commissions [1]	2010		Report	Transformation of nursing education
Lewallen et al. [23]	2011		Discussion paper	Preparation of nurse educators for research & teaching roles
Lindeman [33]	2000		Discussion paper	Transforming nursing education
MacMillan [64]	2013		Report	Future of undergraduate nursing education in Canada
Meacham [90]	2002		Discussion paper	New faculty preparation
Morris et al. [52]	2012		Book	Transformative learning in nursing
NLN [108]	2007		Discussion paper	Nurse educator roles and responsibilities
Nehls et al. [47]	2016		Mixed methods, interviews and database reviews *(n* = 84)	Choosing a PhD program
Oermann et al. [35]	2016		Quantitative survey (*n* = 482)	Roles in nursing programs for PhD & DNP prepared nurses
Schriner [53]	2007		Qualitative, hermeneutical, ethnographic inquiry (*n* = 7)	Transitioning from a clinical expert to a faculty role
Schulman et al. [138]	2006		Discussion paper	Re-examining doctoral preparation
Siler et al. [20]	2001		Qualitative, hermeneutical, phenomenological (*n* = 12)	Understanding the experiences of new faculty
Tanner [96]	2002		Editorial	Advancing teaching pedagogies in nursing education
Barnes et al. [134]	2008	*Mentorship*	Qualitative, in-depth interviews (*n* = 25)	Role of doctoral advisors
Baxley et al. [131]	2014		Book	Mentorship in nursing
Bell-Elliason et al. [139]	2008		Quantitative survey (*n* = 224)	Characteristics of ideal doctoral mentors
Grossman [132]	2013		Book	Mentorship in nursing
Hall et al. [127]	2009		Discussion paper	Mentorship in the development of professional researchers
Johnson et al. [128]	2014		Quantitative, on-line survey (*n* = 95)	How mentoring prepares doctoral students for a faculty role
Noonan et al. [133]	2007		Qualitative, focus groups (*n* = 16)	Mentoring doctoral students
Paglis et al. [129]	2006		Quantitative, survey (*n* = 130)	Mentoring doctoral students as career preparation
Rose [130]	2005		Quantitative, survey (*n* = 537)	The ideal doctoral student mentor
Hoessler et al. [48]	2015	*Graduate TAs*	Mixed methods, document analysis	Graduate student teaching training
Kenney et al. [49]	2014		Qualitative (*n* = 13)	Program structures & practices of graduate training
Love Stowell et al. [41]	2015		Conceptual model	Pedagogical preparation of graduate students
Parker et al. [135]	2015		Qualitative, survey (*n* = 48)	Training for graduate TAs
Agger et al. [18]	2014	*Doctoral Preparation*	Qualitative, semi-structured interviews (*n* = 15)	DNP prepared nurses in academic roles
AACN a [17]	2016		Report	Faculty vacancies – DNP not prepared for academic role
Apold [109]	2008		Discussion paper	Doctoral nursing education
Nyquist et al. [117]	2004		Discussion paper	Re-designing doctoral nursing education
Walker et al. [118]	2016		Discussion paper	Doctoral education for nurses – PhD or DNP?
Winter et al. [97] ^e^	2000		Discussion paper	Evaluation criteria for practice-based doctoral degrees
Acorn et al. [77]	2013	Theme 2: Lack of consensus on scholarship	Discussion paper	Defining and describing scholarship in nursing
Allen et al. [78]	2005		Discussion paper	Differentiating scholarly teaching & the scholarship of teaching
Benigni [115]	2007		Discussion paper	The teacher-scholar
Boyer [12]	1990		Discussion paper	Domains of scholarship
CASN [70]	2013		Report	Scholarship among nursing faculty
Chalmers [68] ^b^	2011		Discussion paper	Recognizing & rewarding the scholarship of teaching in higher education
Chandramohan [38]	2009		Book	Learning to teach in higher education
Cochran-Smith [36]	2003		Discussion paper	Teacher preparation in higher education
Darling-Hammond et al. [45]	2002		Secondary analysis (*n* = 300)	Teacher preparation
Fincher et al. [67]	2006		Discussion paper	Defining and describing scholarly teaching
Gardner et al. [57]	2010		Discussion paper	Teacher-scholar model
Glanville et al. [13]	2004		Discussion paper	Defining the scholarship of teaching
Glassick et al. [71]	1997		Book	Scholarship in the professoriate
Gubbins [66]	2014		Discussion paper	Developing a program of scholarship in teaching & learning
Hatch [72]	2006		Book	The practice of teaching and learning in higher education
Korthagen et al. [32]	2005		Discussion paper	Teacher-educator preparation
Kreber [79] ^a^	2015		Discussion paper	Advancing the scholarship of teaching
Kreber [30] ^a^	2002		Qualitative, Delphi exploratory (*n* = 11)	Consensus on scholarship of teaching
Kreber [28]	2002		Discussion paper	Excellence in teaching & scholarship of teaching should be rewarded
Kreber et al. [42]	2005		Qualitative, exploratory, semi-structured interviews (*n* = 31)	How university instructors learn about teaching
Kreber et al. [58]	2000		Conceptual model	Scholarship of teaching
Kuh et al. [84]	2007		Survey (*n* = 29,444 faculty) and (*n* = 65,633 students)	Teacher-scholar model
Martinez [34]	2008		Discussion paper	Reflective exploration of new teacher educators making the transition into the academy
McKinney [7]	2006		Discussion paper	The challenges of the scholarship of teaching and learning in higher education
McKinney [80]	2013		Book	Intra/interdisciplinary scholarship of teaching and learning
Murray [43]	2005		Qualitative, in-depth semi-structured interviews (*n* = 28)	Experiences of teacher educators in the first 3 years of practice
Nicholls [29]	2005		Discussion paper	Understanding scholarship
Norris [31] ^a^	2000		Discussion paper	Teacher-based knowledge and experience or university research-based knowledge and empirical theory
Oermann [44]	2014		Discussion paper	Scholarship in nursing
O’Meara et al. [69]	2005		Discussion paper	Recognition of all forms of scholarship
Rossetti et al. [27]	2009		Qualitative, Interpretive (*n* = 35)	Educational philosophy of teaching
Shulman [73]	2004		Discussion paper	The scholarship of teaching and learning
Shulman [65]	2000		Discussion paper	The future of the doctorate
Sullivan et al. [82]	2008		Discussion paper	A review of professional education
Trigwell et al. [74]	2000		Qualitative, phenomenological (*n* = 20)	A review of professional education
Vardi et al. [75] ^b^	2011		Discussion paper	Promoting the scholarship of teaching & learning
Zeichner [91]	2005		Discussion paper	Teaching teachers
Austin et al. [114]	2008	Theme 3: Research versus Teaching	Discussion paper	The integration of research, teaching, & learning
Austin et al. [81]	2006		Discussion paper	Preparation in the domains of scholarship other than discovery
Brew [86]	2003		Discussion paper, conceptual model	The relationship between research and teaching
Carter et al. [87]	2011		Qualitative, focus groups (*n* = 8)	The importance of teaching
Campbell et al. [116]	2005		Discussion paper	Re-examining the PhD in Nursing
Chen [88] ^d^	2015		Qualitative, interviews and document analysis (*n* = 20)	Priority of research over teaching
DeCourcy [60]	2015		Discussion paper	Describing excellence in teaching
Diezmann et al. [119]	2015		Case study	Transitioning from research scientist to teacher
Fook [106]	2001		Discussion paper	Integration of theory, practice, & research
Ketefian et al. [4]	2015		Discussion paper	Trends & factors influencing doctoral education
Malcolm [89]	2014		Discussion paper	Research-teaching link in higher education
Marentic Pozarnik et al. [59] ^c^	2015		Case study	Developing excellence in teaching competencies
Matthews et al. [26] ^b^	2012		Quantitative, survey (*n* = 522)	Teaching & research gap implications on the scholarship of teaching and learning
Paulsen [93]	2001		Discussion paper	Relationship between research & the scholarship of teaching
Smeltzer et al. [85]	2015		Mixed methods, focus groups and surveys (*n* = 554)	Impact of teaching on research productivity
Starr et al. [40]	2015		Discussion paper	Inquiry on teaching in higher education
Schonwetter et al. [54]	2015		Cross-sectional survey (*n* = 133)	Faculty development teaching training
Van De Ven et al. [126]	2006		Discussion paper	Engaged scholarship between theory and practice
Williams [76]	2008		Discussion paper	Nursing as an academic profession
Cronin [121]	2003	*Epistemic cultures*	Discussion paper	Requirement for alternate forms of scholarship expression
Georges [125]	2003		Discussion paper	Epistemic diversity in nursing
Knorr Cetina [122]	2007		Discussion paper	Epistemic and knowledge cultures
Mork et al. [123]	2008		Ethnographic case study (*n* = 35)	Conflicts of epistemic cultures
AACN [14]	2016	Theme 4: Lack of consensus on formal education for nurse educators	Report	Academic nursing
AACN [16]	2015		Report	Academic Nursing
AACN [10]	2015		Report	Recommended preparation of nurse educators
AACN [101]	2013		Report	Advancing higher education in nursing
AACN [99]	2010		Report	Transforming nursing education
AACN [9]	2008		Report	Recommendations on educational preference for professoriate in nursing education
Austin [55]	2002		Discussion paper	Faculty preparation
Brar et al. [102]	2010		Discussion paper	Advanced nursing education beyond the Master’s degree
CASN [15]	2011		Report	PhD prepared faculty
Grace et al. [83]	2016		Discussion paper	Preparation of nursing scholars & leaders
Kirkman et al. [105]	2007		Discussion paper	A comprehensive review of doctorates in nursing
Loomis et al. [104]	2006		Qualitative, internet-based, exploratory survey (*n* = 69)	Decision to pursue a PhD or a DNP
Minnick et al. [107]	2010		Survey (*n* = 96)	Capacity in doctoral research nursing programs
NLN [22]	2013		Report	Doctoral preparation for nurse educators
NLN [21]	2002		Report	Recommendations for doctoral preparation of nurse educators
Nyquist [113]	2002		Discussion paper	Revising the current PhD program
Wood et al. [100] ^a^	2004		Report	Doctoral nursing in Canada

Denotes country of publication

^a^Canada, ^b^Australia, ^c^Slovenia, ^d^Taiwan, ^e^United Kingdom

A paucity of literature is available on both the formal academic preparation of, and comprehension of academic nurse educators’ roles [5, 25]. The increased emphasis on the achievement of scholarship in higher education, necessitates some background on areas of scholarship, the scholarship of teaching, current practices of higher education teacher preparation, and factors that have had an influence on the academic nurse educator’s role. The perceived value of the scholarship of teaching varied widely across institutions, and ultimately impacted the academic nurse educator role, [26]. In order to address the purpose of the review, the results of the literature search indicated several resulting themes. These themes framed the organization of this literature review: (1) What is an effective educator? (*n* = 9); (2) What is the current practice for the formal preparation of teachers in higher education? (*n* = 32); (3) How is excellence in teaching described? (*n* = 57) and, (4) What conditions influence or have an impact on academic nurse educator preparation for the responsibilities of their roles? (*n* = 55). The following is a discussion of these resulting themes.

### What is an effective educator?

An academic nurse educator is involved in practice, education and research in both baccalaureate and graduate schools of nursing [14]. The role responsibilities of the academic nurse educator identified by the AACN [14] includes articulating and demonstrating the importance of research through the production of new, nursing-specific knowledge, and identifying the link between clinical nursing practice and education, that ultimately will lead to improved health outcomes. For academic nurse educators to meet these role responsibilities, they require the skills to promote student self- development, technical competence and critical thinking ability [27]. The literature identified a lack of agreement on the definition of an effective educator instead, citing personality characteristics as measures of teaching excellence. Successful or effective teachers for example, are described as having the ability to convey concepts in a meaningful way, and to motivate and encourage critical thinking beyond discipline-specific knowledge [27, 28]. Developing those skills associated with excellence in teaching requires both formal preparation and experience. However, the current practice in higher education is to promote ongoing, informal teaching techniques and tips as the pathway to attaining excellence in teaching, rather than acknowledging the necessity for formal pedagogical knowledge [7, 29].

Students’ perceptions of a teacher’s performance in relation to the success of their learning experience is most often associated with teaching excellence [28]. Student ratings and peer evaluations advance the notion that excellent teachers possess expansive teaching and learning knowledge. However, excellent teachers may not be able to articulate their teaching practice in relation to educational theories [30]. The practice of emphasizing and rewarding outcomes or products in the form of publication or teaching evaluation may be ignoring the process by which faculty learn about teaching. Awards for teaching excellence are generally not made on the level of teaching knowledge [28]. Similarly, then, can one assess the quality of a nurse educator’s teaching effectiveness by virtue of possessing the credential of a PhD?

According to Kreber [28], there is a fundamental difference between *expert* teachers and *excellent* or *effective* teachers. While expert teachers are unfailingly excellent teachers, excellent teachers may or may not be experts. Experts are relentless in their pursuit of new learning opportunities and reflecting on not only on their personal teaching experience, but on how educational theory explains their practice [28]. However, experienced individuals who do not engage in reflective practice are not considered expert teachers [28]. Informal teaching knowledge garnered from personal experience is inadequate as a basis in providing quality education [31]. Thus, effective teachers require a combination of discipline-specific expertise and pedagogical knowledge based on experience and educational theory [28].

In their interpretative study, Rossetti and Fox [27] illustrated the practices of effective teachers. Regardless of the disciplines across which this study was conducted, the findings are relevant to the discipline of nursing as they are central to the nature of professional nursing practice. Effective teaching practice is dependent on a teacher’s formal knowledge of teaching. While nurse educators are considered experts in their area of practice, clinical expertise does not necessarily equate into teaching expertise. Most often those who come to the academy are experts in their fields or disciplines but lack pedagogical knowledge [3]. There is an important distinction between the discipline of nursing and the discipline of education; teaching is both a profession and a second discipline. Therefore, academic nurse educators must develop expertise in pedagogical practices [7, 8]. In order to provide the high quality instruction that is required for new nurses in meeting both current and future health care demands, academic nurse educators need to be highly skilled teachers.

### What is the current practice for the formal preparation of teachers in higher education?

Increased societal demands for the responsibility and accountability of services provided are particularly relevant to the professional disciplines of medicine, law, nursing, and teaching [32–35]. As a result, the responsibility for educating skilled professional practitioners has been entrusted to educational institutions [36]. Providing a high quality education should be central to educators across disciplines; yet, the literature lacks in research on teacher (educator) preparation in higher education [6, 27, 29, 37–39]. In spite of the increased focus on the educational practices of teachers in the delivery of quality education, minimal attention has been afforded to higher education teacher preparation practices and policies [36, 40, 41]. Significantly, there is a finite body of research on teaching expertise in higher education [42].

Institutional culture defines scholarship according to research and publication output, rather than teaching knowledge [34, 43, 44]. Despite the importance of pre-service training needs, the pursuit of traditionally acknowledged scholarly activities are given the highest priority by the academy [45]. For example, utilizing doctoral students ill-prepared for teaching, is the current practice of teacher education in higher education. Earning income is most probably the motivator for doctoral students, rather than striving to be an effective teacher. Many graduate scholarships available to doctoral students are dependent on teaching an undergraduate class, resulting in many doctoral students learning informally about teaching through their teaching assistant (TA) experience. Graduate teaching experience does not equate with the preparation required to develop teaching proficiency [46, 47], indicating the need for doctoral students to have formal teaching education including pedagogical approaches and curriculum development [48–50]. In general, employing TAs in graduate schools is directed towards meeting departmental teaching requirements rather than towards mentoring graduate students in teaching or developing prospective professors [51]. Subsequently, the utilization of inadequately prepared TAs to deliver undergraduate education is under increasing scrutiny.

A cursory search of the Internet identified that many Canadian universities had teaching centers offering basic teaching courses to graduate students and new faculty that included an introduction to teaching program and/or workshops on teaching-specific topics [48, 49]. However, for the most part, participation in these programs are optional. While university teaching centers assist graduate students in learning to be TAs, they do not provide adequate preparation for a faculty role [47].

Due to the economic climate, advanced practice clinicians currently occupy many nursing faculty positions as educational institutions sustain the practice of devaluing academic proficiency and experience [39]. While advance practice clinicians are clinical content experts, they generally have little, if no, previous formal training in adult education [52–54]. The lack of formal teaching education for graduate nursing students and ultimately, being unprepared for a faculty role is a growing concern [25, 39, 55]. As a result, successful transition from a clinical environment into an academic culture for nurses that pursue doctoral education in their discipline, may be difficult [39, 56]. The goal in delivering effective nursing education is not simply in the nursing content knowledge itself, but rather with pedagogical knowledge that engages and informs that knowledge [52].

### How is excellence in teaching described?

The increasing focus on achievement of excellence in teaching in the literature reflects the emphasis on the pursuit of scholarship in higher education. Achieving excellence in teaching requires a high level of individual commitment to move beyond what is already known and provide students with opportunities to develop their critical thinking abilities [57]. It was apparent in the current literature, that there is little documented research about understanding educators’ experiences relative to how knowledge and practice are developed [58, 59]. Up to the present, the focus of the literature has been on the theoretical concepts of teaching expertise, the scholarship of teaching, and teaching excellence rather than on experiential practices [42, 60, 61]. The present review of the literature identifies a significant theme: the experiences of the teaching profession in general, and specifically, the need for further research in addressing the formal preparation needs of teachers in various disciplines [3, 20, 27, 34, 36, 43, 58, 62–64]. Several topics identified in the literature contribute to the discourse on “teaching excellence”: (a) defining scholarship, (b) the scholarship of teaching, (c) pedagogical knowledge, and (d) the characteristics of effective educators. Findings from the literature for each of these sub-themes will be discussed both generally, and specifically within the discipline of nursing.

#### Scholarship

Shulman [65] described a scholar as a consummate professional; an individual that continuously reflects on their practice while ensuring high standards, and who is open to advancing knowledge to others. Conversely, according to Boyer [12], a scholar was an academic whose primary focus was to conduct and publish research, while the practice of imparting and/or applying knowledge became secondary. The proliferation in the 1960s and 1970s of American higher education created a demand for academic professionals, thus *narrowing* the definition of scholarship that still exists in many institutions of higher learning [66]. Consequently, scholars were defined as academics whose priority was to conduct research and publish, in which research demonstrated scholarly work. The assumption was that imparting knowledge to students simply occurred and therefore, was not deemed to be scholarship [12]. The three basic tenets of scholarship are that the work is made public, it is peer reviewed, and that it can be reproduced by others as a means to advance knowledge [65–67]. Importantly, achieving scholarship is dependent on whether others are able to understand, and are agreeable to this knowledge [65]. Therefore, it would seem imperative that the skills and abilities associated with effective teaching and learning practices, and ultimately knowledge delivery, be a priority in the academy.

The scholarship of discovery is recognized by the academy as the optimum pathway leading to new research funds and status. However, standard criteria is lacking in assessing the achievement of scholarship due to the disagreement around the definition of scholarship in general, and specifically, in teaching [72, 73 74, 70, 75, 30, 28, 61, 6].

Discourse around the restricted interpretation and acknowledgement of scholarship in higher education emerged from the seminal works of Boyer [12] and Glassick, Taylor Huber, and Maeroff [68]. Boyer [12] disputed how scholarship was evaluated and rewarded in higher education, according to this narrow definition. As illustrated by his expanded definition of scholarship, Boyer maintained that scholarship is found in all facets of academic life -- discovery, teaching and learning, integration, and application of knowledge [57, 69]. Teaching involves the process of conveying knowledge through the scholarship of discovery, the scholarship of integration and the scholarship of application [12, 57]. Boyer’s model consists of four separate yet overlapping facets of scholarship. This model has been embraced by both the academy and professional organizations, and adapted to discipline-specific contexts.

According to the Canadian Association of Schools of Nursing (CASN) [70] position statement on scholarship, scholarship is defined as the creation, affirmation, amalgamation, and/or implementation of knowledge intended to advance the discipline of nursing. Specifically, discovery as inquiry leading to new knowledge (original research that advances knowledge); teaching as pedagogical inquiry (discovery, integration, and application); application as discipline-specific knowledge expertise guiding professional practice (using new and synthesized knowledge in problem solving); and integration as the synthesis of knowledge. Further, scholarship involves critical reflection, discipline-specific knowledge expertise and innovative approaches to topics of interest under study [67, 71]. These statements expand on Boyer’s [12] traditional definition of scholarship that included discovery, teaching, application, and integration. The question for me, related to the scholarship of teaching, is how will academic nurse educators effectively convey knowledge to nursing students without having a solid pedagogical foundation themselves?

#### The scholarship of teaching

As with scholarship, the difficulty in defining the scholarship of teaching has been cited throughout the literature by scholars from all disciplines [29, 44, 58, 66, 68, 69, 72–76]. Elaborating on the relationship between teaching, scholarly teaching, and the scholarship of teaching, Fincher and Work [67], described teaching as the development and delivery of activities designed to promote learning, while scholarly teaching advances teaching by connecting teaching with learning. Student learning is the outcome of both teaching and scholarly teaching [67]. Scholarly teaching requires ongoing revision of course materials including curriculum development and integration of published research into the course content, critical reflection, and mentoring students [77, 78].

A scholarly approach to teaching involves the application of educational theory and research to practice [58], in which the focus is on process rather than the product. The scholarship of teaching involves the understanding of effective teaching and learning practices that both enhance and expand learning opportunities outside of the traditional classroom experience [67, 77]; it is not a fourth distinct form of scholarship but may involve discovery, integration, or application [67]. However, confounding the problem is that Boyer’s definition of the scholarship of teaching is not clear [13, 44, 67, 77, 79].

Based on this lack of consensus, McKinney [80] emphasized the need to clearly distinguish between teaching and scholarship. She elaborated further, adding that disciplinary differences impacted on how academic activities were defined in relation to scholarly activity and scholarship. Often, scholarly teaching and the scholarship of teaching are used interchangeably. However, in reality, good teaching or teaching excellence is being practiced rather than the scholarship of teaching [67]. While teaching and scholarly teaching facilitate learning, they do not constitute scholarship [77, 78]. Because of this lack of consensus, there is disagreement both between and within disciplines about accepted standards of scholarship and related activities.

Intense competing values within in the academy are responsible for institutional structures and practices to prevail in academic communities and within academic cultures, influencing the values and rewards systems [7, 81]. As a result, the scholar’s role is designated as a researcher in contemporary higher education practice [29, 82], with very little regard to the scholar’s ability as an effective teacher [58, 79, 83, 84].

Boyer [12] and Glassick et al. [71] advanced the theme that in narrowly defining scholarship as it related to discovery, priority consideration and ultimately, significant value was placed on research. Consequently, a reward system defined in totality by research and research-related activities, became the accepted benchmark for achieving scholarship [72, 85]. Therefore, for teaching to be acknowledged as an accepted form of scholarship, its practice must be recognized as discovery of new knowledge [66, 74]. Firstly, if the priority of doctoral education is to prepare researchers, and scholarship is defined in terms of research, to what degree does doctoral preparation advantage academic nurse educators in their teaching roles? Secondly, do these quantifiable measures equate into teaching excellence in nursing education?

The scholarship of teaching is commonly regarded as “*the”* indicator of excellence in teaching, perpetuating the belief that excellent teachers possess extensive pedagogical knowledge [58]; a dilemma faced by many educational institutions in addressing the relationship between teaching and research [68, 75, 86–90]. Teaching excellence is generally measured through demonstrated outputs that include teaching awards, excellent evaluations, and scholarly publications [60, 75, 91, 92]. CASN’s [72] description of achieving the scholarship of teaching (in nursing) validates this practice in which the scholarship of teaching is evidenced by peer reviewed presentations, publications, grants, and other related activities. Oermann [44] argues that there needs to be a broader perspective on how the scholarship of teaching is evaluated. For example, shifting the emphasis from the traditional forms of evidence of achievement to consideration for investigations on effective learning and teaching practices that promote student learning.

#### Pedagogical knowledge

Pedagogical knowledge is knowledge of the principles of effective teaching and learning [58, 93]; it is an essential component of learning to teach [42]. Pedagogical knowledge includes the skill to present discipline-specific content in a way that facilitates understanding and the ability to facilitate critical thinking and self-directed learning [58].

Due to a wide variation in student learning preferences, teachers must have the ability to effectively articulate and execute alternative forms of content delivery. This ability is founded on formal knowledge of pedagogical practices [73]. Pedagogical skills are essential because given the critical enquiry level of students, teachers are required to provide effective instruction for unanticipated and unfamiliar learning situations [73]. Pedagogical content knowledge is the link between content and pedagogical knowledge [93]. In higher education, discipline-specific knowledge and pedagogical knowledge are inextricably connected [43].

The modernization of health care, rapidly increasing technologies, globalization, and the worldwide shortage of nurses implicate the necessity for changes in how nurses practice, and, more importantly, how they are educated. It is imperative that health professionals possess a high level of proficiency in both pedagogical knowledge and teaching skills to effectively meet the demands of current teaching and the health care systems in providing quality education [39, 92]. Past pedagogical practices used to prepare proficient practitioners is outdated. As a result, contemporary teaching and advanced pedagogical theory must inform each other [3, 94–96]. Regardless of the discipline, effective educators including nurse educators, require similar formal knowledge in adult education and pedagogical theory, providing the foundation for teaching practice.

However, due to current perceptions (both organizational and individual) around the value of teaching, and resultant lack of importance placed on teaching is evident throughout institutions of higher learning. For example, while the mission of most higher education institutions is identified as teaching, scholarship, and service, scholarship (research) is generally the priority [11]. It is imperative that agreement on the value of teaching be addressed at both the institutional level and specifically, within the multiple academic communities that constitute the institution.

### What conditions influence the academic nurse educator role?

A PhD requirement, perpetuation of epistemic communities, and a doctoral supervisor’s mentorship role in PhD programs have the potential to impact the nurse educator’s role. These will be discussed in the following section.

#### PhD requirement

The origin of the word “doctorate” is from the Latin verb docere “to teach.” Historically, the doctorate was acknowledged on the premise that teaching was both an honor and a rare opportunity [97]. Despite etymology, doctoral programs across disciplines are generally designed to provide a research-intensive training experience [11, 98, 99]. Doctoral education is intended to produce scholars who will advance the discipline. Doctoral students are given the opportunity to develop their expertise in order to carry out original research and scholarly inquiry that leads to new discipline-specific knowledge [11, 100]. However, for future faculty to be successful in the academy, graduate programs need to expand their educational approach beyond research training, to include all role responsibilities, and significantly, to recognize the importance of teaching [62].

A PhD is the academic requirement for most tenure-track nursing faculty positions, yet most notably recognized as a research degree [101–104]. Numerous professions have responded to the demand for increased academic ranking by requiring a doctoral degree [103, 105]. For the discipline of nursing to be acknowledged and accepted on the same level as other professions is the appeal of the PhD [105]. Fook [106] identified that when professional knowledge is validated according to patriarchal criteria, those professions achieve position and status among other professions. Accordingly, those individuals with a PhD designation are considered to be privileged scholars [65].

Preparation at the doctoral level is a requisite for academic nurse educators in order to make a contribution of new knowledge the body of nursing literature and to prepare future nurses [1, 3, 94]. Most doctoral programs in the United States identify the PhD as a research degree and the advanced practice degree as a DNP [107]. Interestingly, a PhD has become the both a required and preferred credential for a teaching position in many universities. Clearly, research has taken precedent over teaching.

However, heavy teaching loads are often assigned to new faculty that have recently completed their PhDs. In general, academic nurse educators are involved with teaching and teaching related-activities for an estimated 27 h on a weekly basis [108]. Significantly, while teaching generally occupies the majority of faculty time, it is a role for which they are not adequately prepared [11, 109]. Interestingly, the first doctoral degrees earned by nurses were in education with a teaching focus [6]. Formal teaching preparation should be fundamental to doctoral training because of the emphasis on job-related teaching that many doctorally prepared individuals encounter [46, 94, 110]. Thus, if we as academic nurse educators have valid rationale for requiring a PhD, then it is the responsibility of doctoral programs to provide the knowledge and skills necessary to educate nursing students, and ultimately future nurses [2, 11, 16, 55, 111–113].

Preparing professionals to conduct academic research that contributes to new knowledge is a well-established practice throughout PhD program curricula [7, 55, 114–118]. Although original research is central to both the academy and the public, the relationship between doctoral education and actual job expectations have become largely disconnected [18, 114, 116, 117, 119, 120]. This disconnection has brought into question the overemphasis on research and ensuing lack of teaching mentorship in doctoral programs [11, 18, 114]. Most doctoral programs lack a systematic approach to preparing doctoral students as educators for the transition to the faculty role. Most doctoral curricula do not include structured teaching experiences resulting in the lack of any formal pedagogical training [11]. Because curricular content is left to the discretion of individual institutions (and based on their perception of the value of teaching) there is no real commitment or recommendation on how best to educate future nursing professoriate [11].

In acknowledging that the capacity to carry out research is not synonymous with being an effective teacher, the AACN [9] recommended the need for formal pedagogical preparation including teaching practicums in doctoral programs.. Interestingly, a task force report [99] identified teaching as a substantial component of many PhD graduates’ roles, yet simultaneously indicated that PhD preparation was directed towards a research career. According to Brightman [2], PhD programs do not offer formal training in teaching because of the traditional practices perpetuated by the academic system in which research is overvalued and teaching undervalued; thus, the emphasis of doctoral programs is on learning research methods and discipline-specific knowledge. How is the capacity to conduct research equivalent to teaching proficiency?

#### Epistemic cultures

It is evident from the manner in which most professional communities function, that there is a great discrepancy as to the meaning of the scholarship of teaching and related activities. The very organization of these professional communities which are framed around discipline-specific theoretical knowledge, clinical practices and related reward systems lends itself to the perpetuation of an epistemic culture [121]. Epistemic cultures are self-contained in relation to the regulation of membership, policies, procedures and practices [121–123]. For example, groups of practitioners and groups of researchers constitute different epistemic cultures. Importantly, these practices are implicated in widening the theory practice divide [122, 124–126].

Specific knowledge processes and practices are embedded within these groups and reinforced by organizational and/or institutional context that results in barriers that impede the integration of knowledge across practices. Within epistemic communities, discipline specific knowledge is both developed and sustained by the community members, thus facilitating isolated and limited interactions both within, and outside of the community [123, 126]. Because embedded processes and practices within epistemic communities are reinforced by organizational and/or institutional context, there is a great likelihood for barriers to develop, creating a challenge for successful/effective involvement and/or participation from other professional communities [123]. This is significant in relation to the current practices of the nursing profession in seeking collaborative status within an interdisciplinary context.

Knorr Cetina [122] described the focus of an epistemic culture to establish the perpetuators of knowledge construction rather than of knowledge construction itself. For example, one of the most common practices of academic epistemic cultures is to disseminate knowledge at conferences attended by those within the same culture which further reinforces existing practices and preferences [121]. Ultimately, professional silos are created and maintained, facilitating isolated professional practices and encouraging competition among these professions, otherwise known as tribalism [1, 121]. Due to the continued reinforcement of traditional practices within epistemic communities, an expanded view of teaching must be both introduced and integrated into the broader academic community [7]. The academic nurse educator role has been significantly impacted by the ability of epistemic cultures to both flourish and sustain their practices across academic and nursing communities, and within PhD program curricula. Importantly, these long standing traditions within disciplines are highly influential on the focus for graduate studies [81].

#### The role of mentoring in doctoral programs

Because the mentor-mentee relationship has received increased attention in relation to its critical role in graduate education, there has been a recent proliferation of literature on the subject. The mentor-mentee relationship has advanced from simply seeing a student through to degree completion to actually influencing a student’s professional and personal growth [49, 127–130]. Currently however, mentoring relationships in doctoral programs most often focus on the pursuit of scientific inquiry, the transferring of knowledge, facilitating research activities and developing research partnerships [131]. Because mentorship impacts significantly on the development of the doctoral student, teaching, research, and academic role responsibilities should be included [56] as essential components of this relationship.

A mentor is described as a successful leader who advises, coaches, role models and initiates professional connections [132]. The mentor is considered a role model from which the mentee mirrors their mentor’s demonstrated behaviors and practices as part of their own professional working identity [128, 132]. This psychosocial component of mentoring contributes positively to building the mentee’s level of confidence and ultimately, competence [128]. Importantly, mentoring perpetuates itself, in that graduates of doctoral programs who experienced positive mentoring relationships have the potential for, and the willingness to, mentor others [133].

Responsibilities attached to modeling with doctoral students includes both initiating and facilitating informal and formal dialogue and structured, discipline-specific learning sessions around professional roles and responsibilities [81]. The purpose of these activities are to support the student as they develop their own identity as both a scholar and a member of a profession [81]. Additional responsibilities include advising students, evaluating or providing feedback to colleagues, administrative duties, and developing new technology and approaches to teaching [55].

Barnes and Austin [134] described mentorship as the development and maintenance of an effective working relationship throughout the entirety of a student’s doctoral studies. Campbell et al. [116] described mentoring as a relationship between that mentor and mentee that is individualized to meet both the professional and personal goals of the student. Necessary activities to be included throughout this relationship would be to provide advice, information and constructive feedback, to ensure high research standards, to facilitate professional networks, and to introduce the student to discipline-specific practices. According to Barnes and Austin [134], research and research-related activities are both regarded and rewarded more favorably at research universities than teaching and teaching-related activities. Granting agencies policies, and teaching and research, publishing, and applying for external funding requirements were identified as limitations to mentoring by supervisors [134]. As a result, teaching mentorship was not regarded by doctoral supervisors as a responsibility of their role.

## Discussion

The requirement of a doctoral (PhD preferred) degree for academic nurse educators, the perpetuation of epistemic communities, and the lack of effective mentoring in doctoral programs, have greatly impacted the roles and responsibilities of academic nurse educators. Specifically, the need to legitimize professional knowledge, reinforcing traditional practices within professional communities, and research only mentorship, do not meet the realistic expectations of the role of the academic nurse educator in the delivery of nursing education. However, the current literature identifies these same issues [8] of which there has been no substantial change.

Several key themes were identified from the review of the literature. The expectations of academic nurse educators in that they are required to deliver a quality education to nursing students, yet most have no formal preparation. A doctoral degree (PhD preferred) is the requirement for a position as an academic nurse educator in most university schools of nursing, with the major portion of a nurse educator’s workload is teaching and related activities. However, a PhD is generally research-focused with no formal, organized pedagogical courses or experiences for doctoral students. There is an inability both across and within disciplines to reach consensus on what constitutes scholarship from which standards for excellence in teaching, scholarly teaching, and the teacher-scholar vary widely. Due to this lack of consensus in interpretation, interdisciplinary collaboration will continue to struggle. Institutional emphasis and ultimately the reward system, is based on research related productivity, while teaching is perceived to be secondary. Epistemic cultures within the university (academic) community further perpetuated the research versus teaching debate. Future discussion should consider the *recommendations* of national nursing licensing bodies versus the desired future of nursing education based on realistic expectations. Rather than making recommendations of which institutions may choose or not choose to follow, there needs to be a consistent approach to the preparation of academic nurse educators. Other related issues include the lack of consensus regarding educational preparation and incongruences between the desired future of nursing education and current graduate nursing curricula. Might graduate programs offer streams in education, research, and/or clinical practice that are designed to meet specific roles and responsibilities?

However, several practices unique to nursing are implicated in the lack of progress in the delivery of profession nursing education. For example, registered nurses in practice, with or without a bachelor’s and/or master’s degree in nursing, may potentially supervise undergraduate students in a clinical and/or laboratory setting. Registered nurses with a PhD should supervise graduate students, however, this does not happen in practice areas where the practice *expert* does not have a doctoral degree. While the emphasis remains on educating specified numbers of undergraduate nursing students to meet the needs of health care systems, there is not the same focus on consistent preparation of graduate nursing students at the doctoral (PhD) level; these are the very individuals that the nursing profession needs to be effective educators. A particular gap in the literature is the number of research-based studies (*n* = 33) that were identified in the literature search. It is evident that there is a lack of evidence-based research and the need for studies to be undertaken regarding the most effective preparation for academic nurse educators.

## Conclusion

This paper explored the state of the literature regarding doctoral (PhD) preparation of academic nurse educators; 139 works have been synthesized to meet the aims of the literature review. Given the current doctoral (PhD) curricula both in Canada and the United States, adequate preparation for the role of academic nurse educator in effectively meeting related responsibilities remains in jeopardy.

## Abbreviations

AACN: American association of colleges of nursing
CASN: Canadian association of schools of nursing
DNP: Doctor of nursing practice
EdD: Doctorate of education
NLN: National league for nursing
PhD: Doctorate of philosophy
